# Supplementary material for: Light-switchable propulsion of active particles with reversible interactions
Source: Nat Commun. 2020 May 26;11:2628. doi: 10.1038/s41467-020-15764-1 (PMC7251099; doi:10.1038/s41467-020-15764-1)
Supplement: Supplementary file 1 — Supplementary Information [file 41467_2020_15764_MOESM1_ESM.pdf]

Supplementary Information

Light-switchable propulsion of active particles with reversible interactions

Vutukuri et al.

Supplementary Note 1: Particles synthesis

TiO_2 particles were synthesized following the procedure of He *et al.*[1]. Briefly, 2.66 mL of titanium (IV) isopropoxide ($Ti[OCH(CH_3)_2]_4$, 99.999%, Sigma-Aldrich) and 0.70 mL formic acid ($\geq$95%, Sigma-Aldrich) were rapidly dissolved in 60 mL of absolute ethanol ($\geq$99.8%, Sigma-Aldrich). Next, the reaction mixture was stirred (500 rpm) for 5 min and then transferred to a Teflon autoclave. The autoclave was subsequently kept in a pre-heated oven at 150°C for 6 h. The reaction mixture was cooled down to room temperature in ambient air. After synthesis, the resulting TiO_2 particles were cleaned several times with ethanol and MQ water (18.6 MΩ.cm) to remove the secondary nucleation and unreacted chemical species. The size and polydispersity of particles obtained using scanning electron microscope, and static light scattering. The size of the particles was 3.5 μm with a size polydispersity of 9 %. Passive silica particles (Microparticles, Gmbh, Germany) of the size 2.1 μm with polydispersity of 3 % were used in active and passive mixtures study.

We used thermal sintering method to convert amorphous TiO_2 particles to anatase phase. The dried sample was thermally sintered using an electric furnace (Nabertherm Co., Germany) at 400°C at the ramping rate of 75°C/min for 2h. Prior to the X-ray powder diffraction (XRD) measurement the sample was dried at ambient room temperature. XRD pattern confirms that anatase phase of thermally sintered TiO_2 spheres as shown in Supplementary Figure 1. XRD of anatase TiO_2 were measured using a X'Pert Pro (PANalytical B.V., Netherlands) powder diffractometer equipped with Cu Kα radiation (45 kV, 40 mA) operating in reflection mode. We measured the surface potential of the active and passive particles using Malvern Zetasizer Nano. The surface potential of half-gold coated sintered TiO_2 particles was $\zeta_{\text{active}} \approx -39$ mV, and $\zeta_{\text{passive}} \approx -48$ mV for passive particles. To maintain the same surface conditions of the cover-slip before and after the fixation of the swimmer in the active and passive mixtures experiments, we attached the half-Au coated anatase TiO_2 particles by simple drying of the

particles from a dilute dispersion.

Supplementary Note 2: Fabrication of self-propelled particles

To obtain half-Au coated anatase TiO_2 particles, first a monolayer of particles was deposited on a glass slide. The glass slide was rinsed several times with acetone and ethanol, and subsequently plasma-cleaned for 30 s at high RF level (Harrick Plasma, Ithaca, NY). The monolayer of particles was obtained by depositing a 100 μL dispersion of 0.8 wt $\%$ particles on the slide using a spin-coater (model WS-400B-6NPP Lite, Laurell Technologies, North Wales, PA) in a three-step program (30 s at 500 rpm, 70 s at 1200 rpm, 20 s at 4500 rpm). Gold was then sputter-coated (Safematic, CCU-010 HV Compact Coating) over the monolayers to get a final thickness of $\sim$40 nm. Finally, particles were detached from the glass slide by sonication (Bandelin, Sonorex) for 10-15 min and collected by sedimentation. Hybrid TiO_2-Ni-Au particles were used to control the cap orientation using external magnetic field, in this case, a Ni layer was deposited and sandwiched between the TiO_2 and gold.

Supplementary Note 3: Imaging and data analysis

We followed the particle dynamics using an inverted microscope (Zeiss 200M) with a high numerical aperture oil objective lens (60x 1.25NA, 100x 1.3NA, Oil, EC Plan NeoFluar) and equipped with a mercury lamp (X-Cite 200 DC, Excelitas Technologies, USA), and a Nikon inverted fluorescence microscope equipped with a CCD camera (Olympus CKX41). Light was sent through the microscope objective lens and the intensity of light was uniform across the area of interest. Moreover, the light source was equipped with a manual knob to control the intensity of light. Different bandpass filters were used to obtain light emission in 370-385 nm (UV), and 535-565 nm (green). We used Lab-Tek cover-glass (Thermo Fisher Scientific) chambers to follow the dynamics. In a typical experiment we used 100 μl of dispersion with known particle concentration and the fuel concentration. With our current experimental setup we achieved

time required for switching between two wavelengths is about 0.4 s. This is fast enough for the current study, as the time scale of switching needed to be smaller than the persistence time ($\approx$ 35 sec, $1/D_r$, rotational diffusion of the self-propelled particle) for the self-propulsion. The intensity of light emission was measured with a photo detector (Thorlabs, PM 100D). We acquired images with a frame rate in the range between 5-50 frames/s. The brightness and contrast of the images were adjusted using ImageJ. The particle's centroid was extracted from time-lapsed images using ImageJ plugin, Trackmate.[2]

Supplementary Note 4: The effect of cap orientation

To investigate the effect of the gold-cap orientation of the active particles and the resulting flow fields on the clustering behaviour of active and passive mixtures, we sandwiched a Ni layer between the gold layer and the surface of $\mathrm{TiO_2}$ particle such that the orientation of particle can be controlled using a magnetic field as illustrated in the Supplementary Figure 7. We apply the magnetic field (0.05 T) to change the cap orientation of the particle while it propelled from perpendicular (90°) to parallel (180°) orientation with respect to the substrate, and thus generating a net thrust by the phoretic flow pointing towards the wall. Hence, the active particles are pushed away in all the directions as shown in Supplementary Figure 7a-b, whereas the passive particles are only pushed along the propulsion path of the active particle as shown in Supplementary Figure 7c.

Supplementary Note 5: Phoretic flow due to an isotropic sphere in proximity to a wall

We follow the continuum framework of phoretic motion and consider the flow generated between two surfaces $\mathcal{S}_1$ (a flat no-slip wall) and $\mathcal{S}_2$ (a spherical particle), in a space filled by a fluid of dynamic viscosity η and density ρ, containing a solute species of local concentration C and diffusivity D. The chemical activity $\mathcal{A}$ quantifies the constant rate of solute release ($\mathcal{A} > 0$)

or absorption ($\mathcal{A} < 0$) at the surface

$$D\boldsymbol{n} \cdot \nabla c = -\mathcal{A} \text{ on } \mathcal{S}_j, \qquad j = 1 \text{ or } 2, \tag{1}$$

with $\boldsymbol{n}$ being a unit vector normal to the surface. Because of the short-range interaction of solute molecules with the boundary, local concentration gradients induce pressure forces that drive the motion of the fluid. Assuming that the interaction layer to be thin, the classical slip-velocity formulation may be used, and the local solute gradients result in a net slip velocity

$$\boldsymbol{u} = \mathcal{M}(\mathbf{1} - \boldsymbol{nn}) \cdot \nabla c \text{ on } \mathcal{S}_j, \qquad j = 1 \text{ or } 2, \tag{2}$$

that drives the flow. Here, $\mathcal{M}$ is the local phoretic mobility of the surface of the particle. Uniform concentration on the flat wall results in the classical no-slip boundary condition there ($\boldsymbol{u} = \mathbf{0}$). When the Péclet number $\mathrm{Pe} = \mathcal{U}\mathcal{R}/\kappa$ is small enough (e.g. small solute molecules), the solute dynamics are governed by the Laplace's equation

$$\nabla^2 c = 0. \tag{3}$$

Here, $\mathcal{R}$ denotes the radius of $\mathcal{S}_2$ chosen as characteristic length, and $\mathcal{U} = |\mathcal{A}\mathcal{M}|/\kappa$ is the characteristic phoretic velocity generated along $\mathcal{S}_2$. When inertial effects are negligible (i.e. $\mathrm{Re} = \rho\mathcal{U}\mathcal{R}/\eta \ll 1$), the flow and pressure fields satisfy the incompressible Stokes equations

$$\nabla \cdot \boldsymbol{u} = 0, \quad \eta\nabla^2\boldsymbol{u} = \nabla p. \tag{4}$$

The diffusive problem for the solute thus effectively decouples from the hydrodynamic problem and may be solved independently. Its solution for the concentration c can be used to compute the slip flow along $\mathcal{S}_2$, Eq. (2), which then determines the flow field by solving Eq. (4).

The problem is non-dimensionalised as follows: with $\mathcal{R}$ and $\mathcal{U}$ chosen as characteristic length and velocity respectively, the characteristic concentration scale is set by $|\mathcal{A}|\mathcal{R}/\kappa$ and the characteristic pressure reads $\eta|\mathcal{A}\mathcal{M}|/\mathcal{R}\kappa$. The resulting calculated solute concentration field

around an isotropic TiO2 particle in proximity to a wall, and streamlines of the corresponding hydrodynamic flow field as shown in Supplementary Figure 6a.

Formulation of the non-dimensional problem

The flow region $\mathcal{D}$ is enclosed between a flat no-slip surface $\mathcal{S}_1$ and the particle surface $\mathcal{S}_2$. The diffusion problem for solute concentration reads

$$\nabla^2 c = 0 \text{ in } \mathcal{D}, \tag{5}$$

$$c = 0 \text{ on } \mathcal{S}_1, \tag{6}$$

$$\boldsymbol{n} \cdot \nabla c = -A \text{ on } \mathcal{S}_2, \tag{7}$$

with the dimensionless activity $A = \mathcal{A}/|\mathcal{A}|$, and the associated hydrodynamic flow problem may now be formulated as

$$\nabla^2 \boldsymbol{u} = \nabla p \text{ in } \mathcal{D}, \tag{8}$$

$$\boldsymbol{u} = \boldsymbol{0} \text{ on } \mathcal{S}_1, \tag{9}$$

$$\boldsymbol{u} = M(\boldsymbol{1} - \boldsymbol{nn}) \cdot \nabla c \text{ on } \mathcal{S}_2, \tag{10}$$

with $M = \mathcal{M}/|\mathcal{M}| = \pm 1$ the dimensionless mobility.

The Stokes flow problem may be conveniently formulated in bi-polar coordinates using the stream function $\psi(\tau, \sigma)$. The stream function is related to the flow vorticity by $\omega = -\nabla^2 \psi$. Since the vorticity $\boldsymbol{\omega} = \nabla \times \boldsymbol{u}$ is harmonic, $\nabla^2 \boldsymbol{\omega} = 0$, we conclude that ψ satisfies the biharmonic equation

$$\nabla^4 \psi = \nabla^2 \nabla^2 \psi = 0. \tag{11}$$

Both the Laplace's and biharmonic equations are solved in bi-polar coordinates. The solutions have the form of series representations. Exemplary calculations for phoretic flows in this geometry may be found in the past work.[3,4]

Supplementary Figures

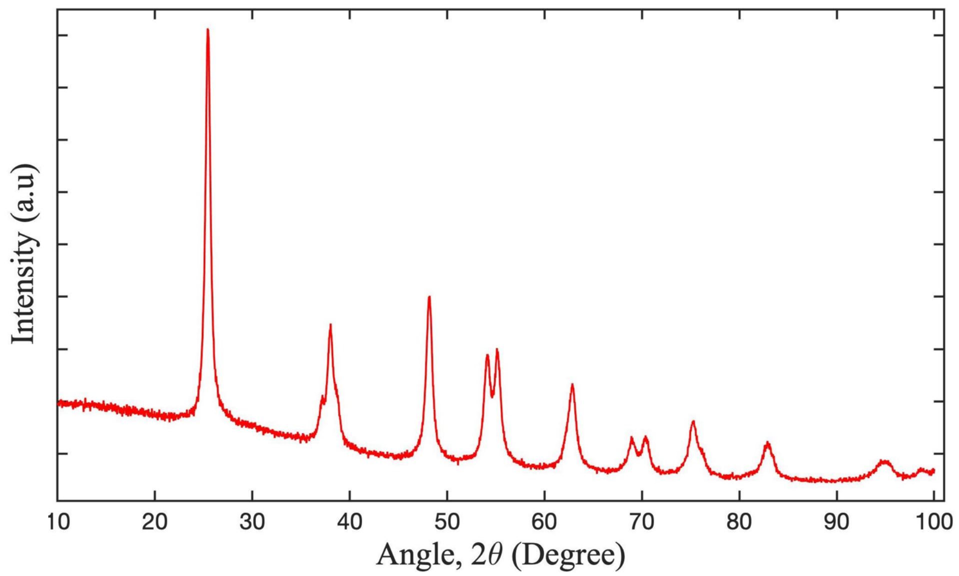

Supplementary Figure1: XRD pattern confirms the thermally sintered TiO_2 particles are in anatase phase.

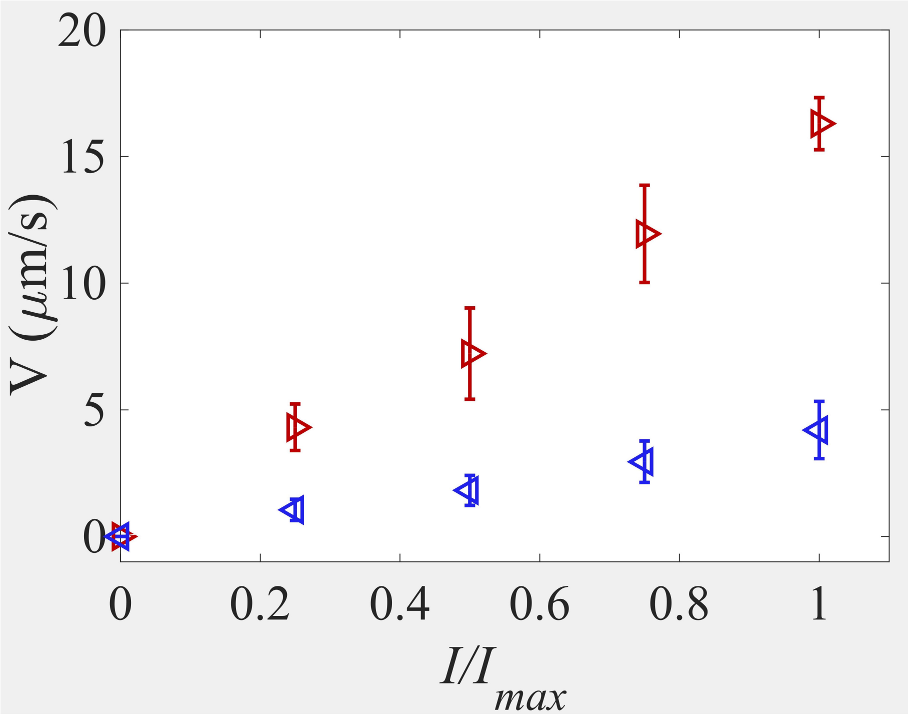

Supplementary Figure2: Propulsion speed of active particle under different light intensities. $I_{max}$is the maximum light intensity (3.5 mW/cm^2) used in our study. Red and blue points represent the forward and backward motions, respectively.

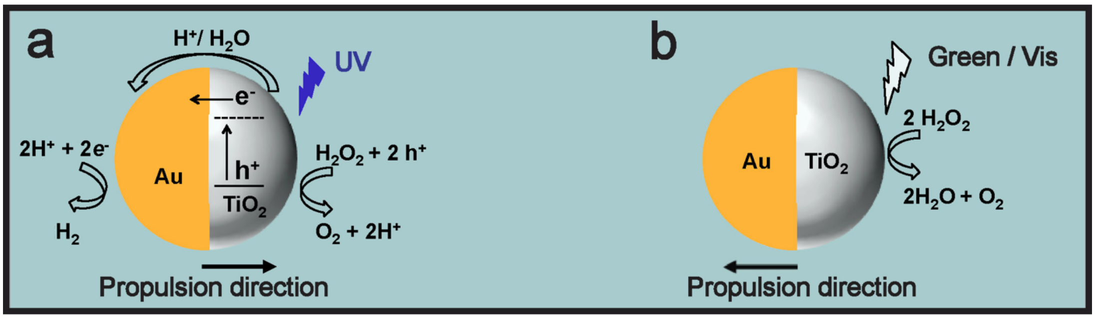

Supplementary Figure3: Schematics of qualitative underlying mechanisms for the particle propulsion direction reversal. (a) under the UV illumination, and (b) green or high intensity visible light irradiation.

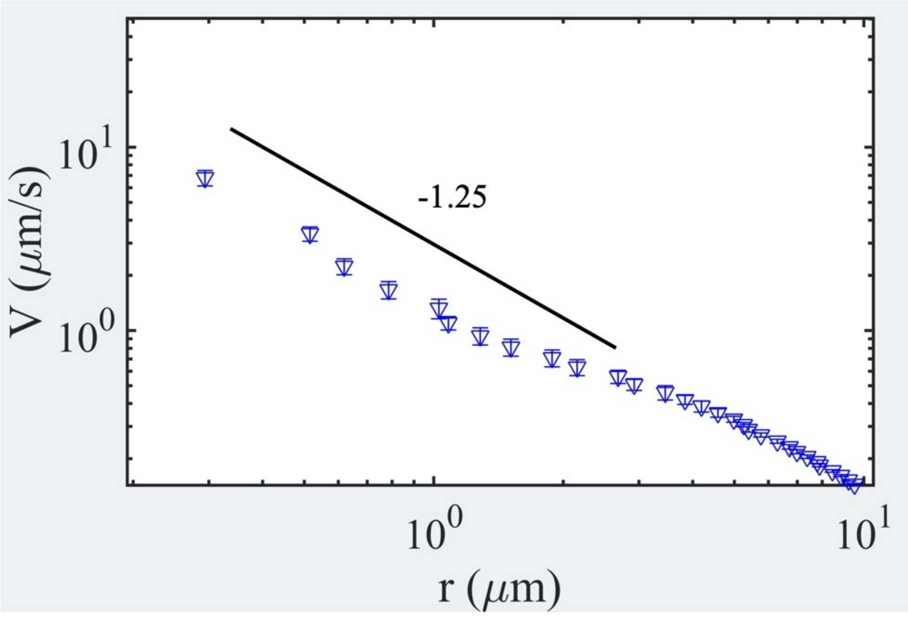

Supplementary Figure4: Spatial dependence of the average velocity of single particles approaching the cluster under the green light illumination.

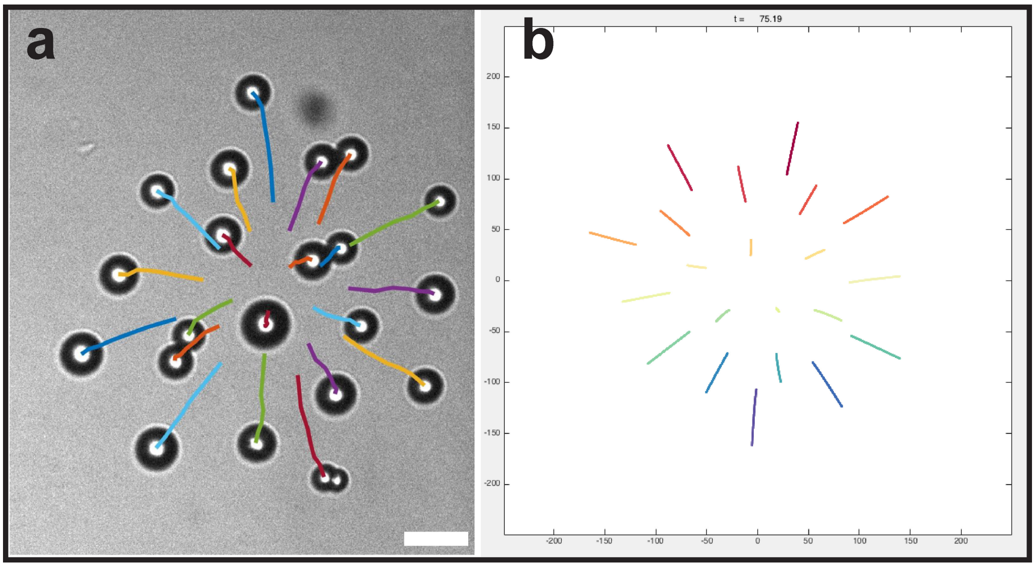

Supplementary Figure5: (a), Flow streamlines revealing the pushing behaviour of isotropic TiO_2 particles under UV light. (b), Simulations shows the similar trajectories for starting with the same initial positions of the particles in the experiments. The scale bar is 5.0 μm.

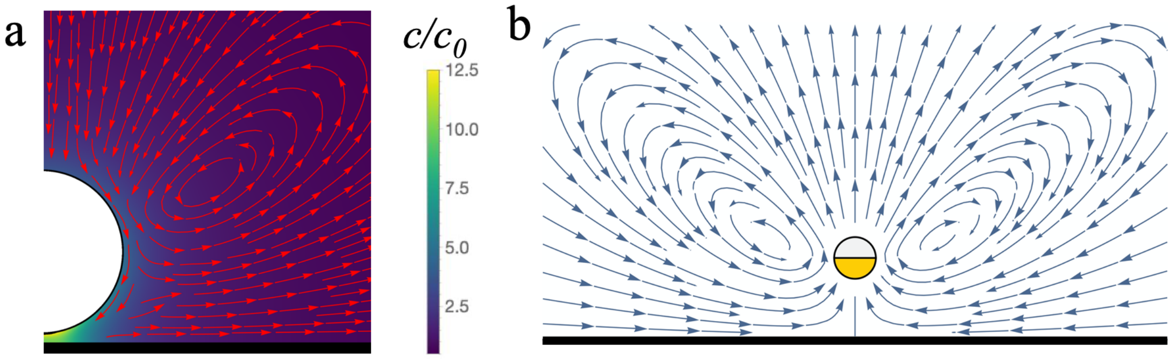

Supplementary Figure6: (a), The calculated solute concentration field around an isotropic TiO_2 particle in proximity to a wall, and streamlines of the corresponding hydrodynamic flow field. (b), Flow streamlines due to a Stokeslet (point force) perpendicular to a no-slip wall. The sucking effect in the plane of the wall dies out with the distance d as $1/d^4$.

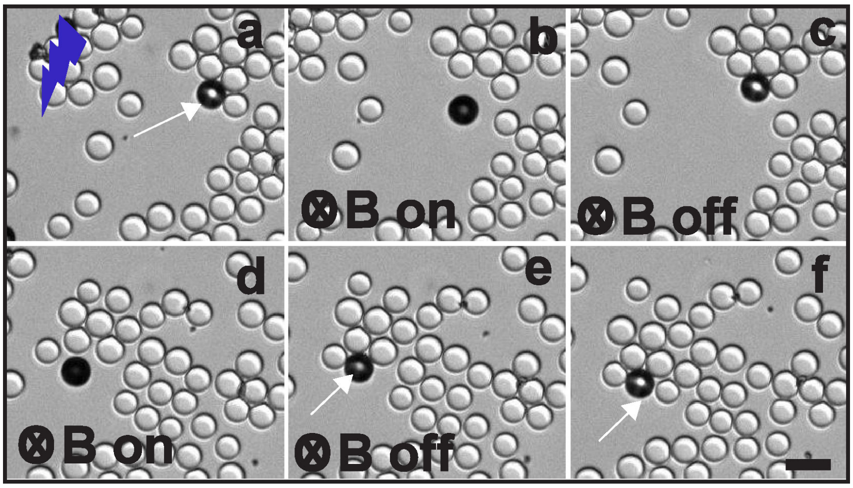

Supplementary Figure7: Investigating the role of the orientation of the gold cap in the clustering behaviour of the active and passive mixtures. (a-f), UV-light is on. (b, d) The magnetic field is on, the Ni-coated side (gold cap) of the particle turned perpendicular to the image plane and the passive particles subsequently pushed away. Magnetic field is applied perpendicular to the image plane. (e-f), Magnetic field is off. White arrow depicts the TiO_2 (non magnetic) side of the particle.

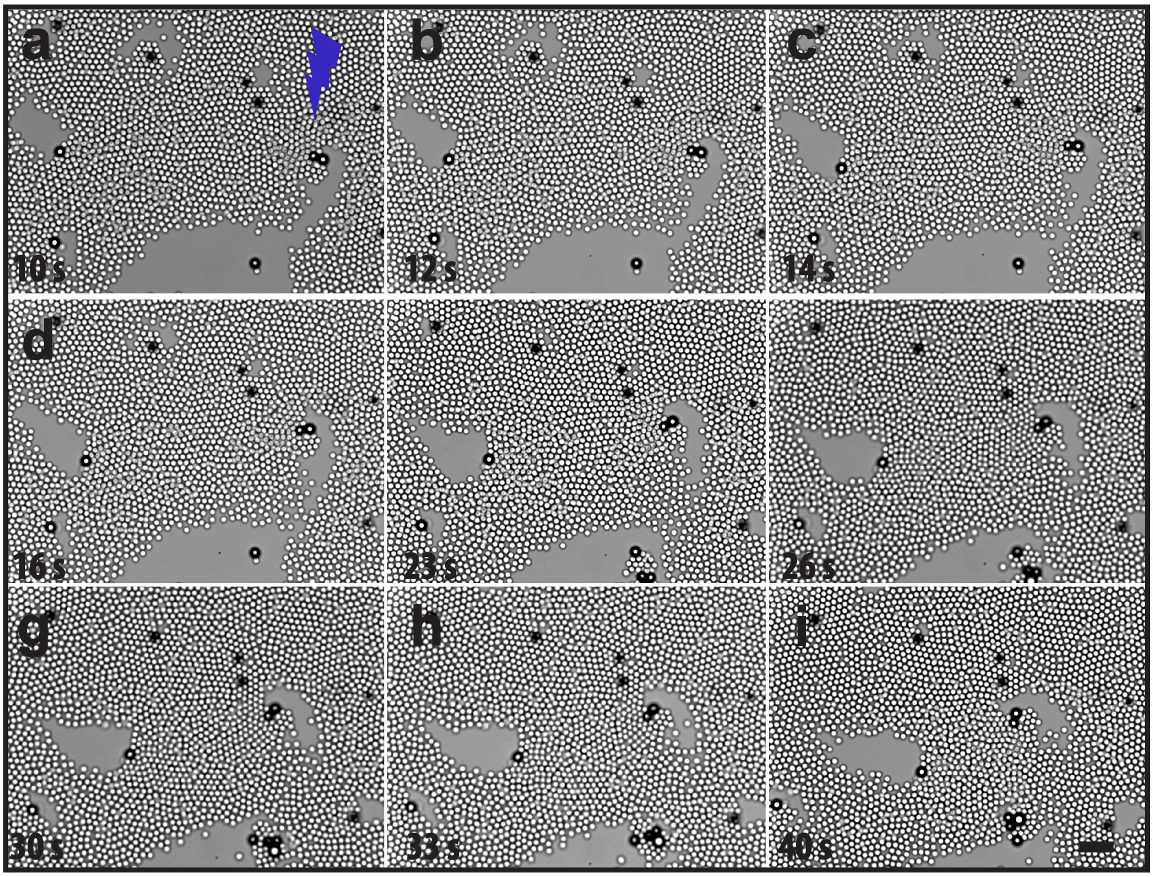

Supplementary Figure8: Active and passive mixtures under UV illumination. (a-i) Time-lapsed bright field images reveal the compression of passive particles by active particles. The scale bar is 5.0 μm.

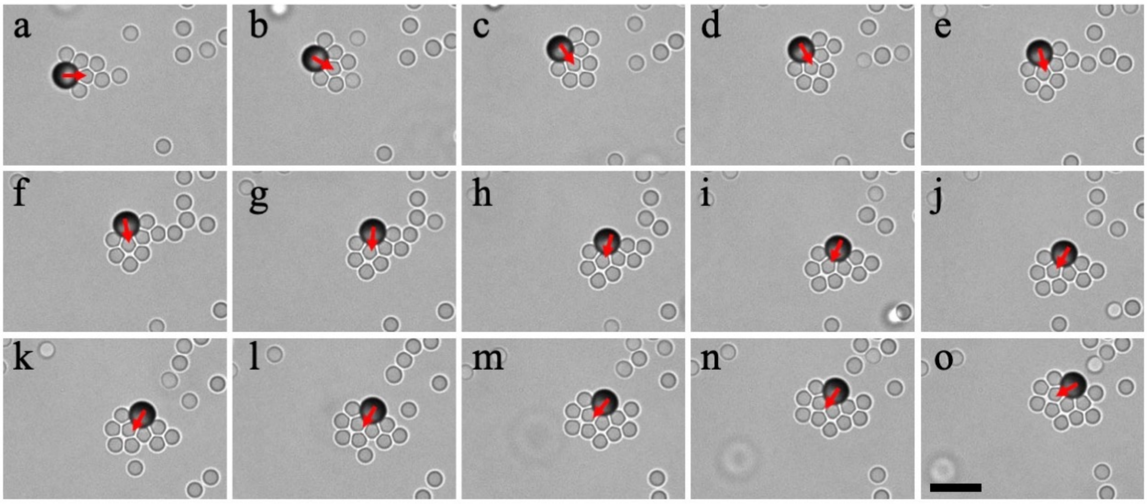

Supplementary Figure9: (a-o),Time-lapsed bright field images showing the propulsion direction with respect to the cap-orientation in active and passive mixtures. Time difference between the subsequent images is 2 sec. The scale bar is 5.0 μm.

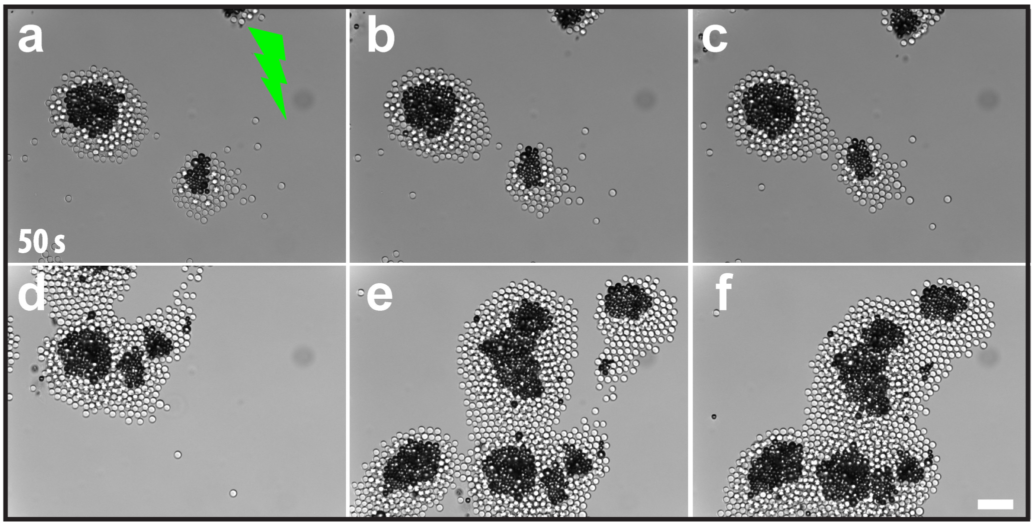

Supplementary Figure10: Phase separation of active and passive mixtures. (a-f), Dynamic clustering and phase separation of the active and passive mixtures. The scale bar is 5.0 μm.

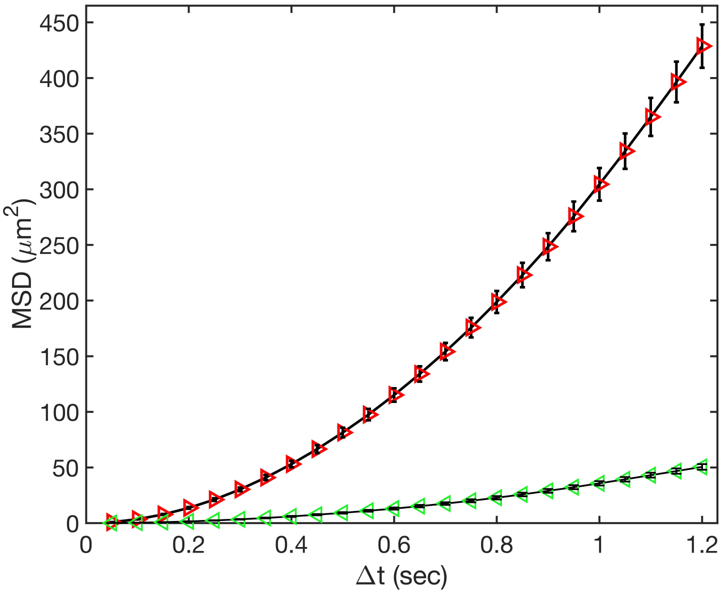

Supplementary Figure11: Mean-squared displacement (MSD) of Janus Au $\mathrm{TiO_2}$ spheres. The solid line is the fit from the MSD equation ($\langle \Delta r^2 \rangle = \langle [r(t+\Delta t) - r(t)]^2 \rangle = v^2t^2 + 4D_t t$). Red colour represents the forward motion and green colour depicts the backward motion of the active particle.

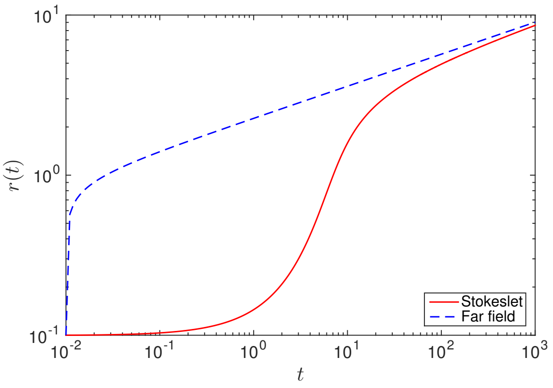

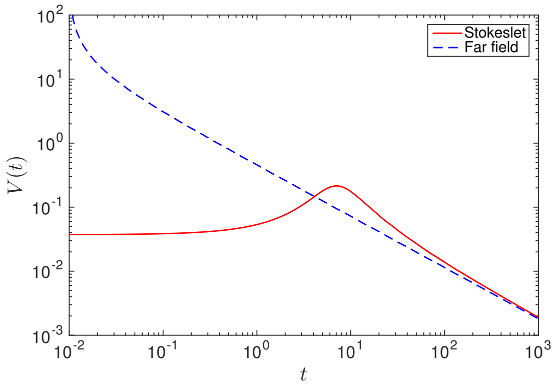

Supplementary Figure12: Comparison of planar trajectories (left) and velocities (right) of a test particle starting at $r_0 = 0.1$ and advected away by the flow due to a point force held fixed above a planar wall. Red solid curves correspond to the Stokeslet flow field, while the blue dashed ones derive from using only the asymptotic r^{-4} term in equation ($\frac{\mathrm{d}r}{\mathrm{d}t} = \frac{r}{(r^2+4)^{5/2}}$).

References

[1] K. He, G. Zhao, G. Han, *Cryst. Eng. Comm.* **16**, 7881 (2014).

[2] J.-Y. Tinevez, *et al.*, *Methods* **115**, 80 (2017). Image Processing for Biologists.

[3] A. Domínguez, P. Malgaretti, M. N. Popescu, S. Dietrich, *Phys. Rev. Lett.* **116**, 078301 (2016).

[4] M. Lisicki, S. Michelin, E. Lauga, *J. Fluid. Mech.* **799**, R5 (2016).
