# Supplementary material for: Light-switchable propulsion of active particles with reversible interactions
Source: Nat Commun. 2020 May 26;11:2628. doi: 10.1038/s41467-020-15764-1 (PMC7251099; doi:10.1038/s41467-020-15764-1)
Supplement: Supplementary file 2 — Description of Additional Supplementary Files [file 41467_2020_15764_MOESM2_ESM.pdf]

Description of Additional Supplementary Files

File Name: Supplementary Movie 1
Description: Time-lapsed overlaid bright-field microscopy movie shows the propulsion direction switchable half-gold coated anatase TiO_2 particle by light modulation. Active particle shows forward motion in the direction of TiO_2 side under the UV illumination, whereas the particle shows backward motion in the direction of gold-coated side under the green light illumination. The propulsion direction reversing is achieved by switching light illumination from UV to green, and vice-verse. The brightness and contrast is adjusted using ImageJ. The movie is accelerated 2X.

File Name: Supplementary Movie 2
Description: Time-evolution of the reversible fusion and fission dynamics of photo-switchable active particles. Under green light illumination, all active particles cluster into a single cluster. This single cluster disintegrates into small clusters when the propulsion direction is reversed by UV illumination. The movie is accelerated 3X.

File Name: Supplementary Movie 3
Description: This movie demonstrates the switchable nature of interactions between the active and passive particles from repulsive to attractive by light modulation. The dynamic response of passive particles to the disturbance velocity flow fields driven by immobilized active particle. The movie is accelerated 3X.

File Name: Supplementary Movie 4
Description: This movie demonstrates the inter-play between attractions and repulsions thus the dynamic assembly and dis-assembly of passive particles in active and passive mixtures. The concentration of active particles is $\phi_a < 0.5\%$ in a concentrated dispersion ($\phi_p \approx 12\%$) of passive particles of $\sigma_p = 2.1$ μm. The movie is accelerated 2X.

File Name: Supplementary Movie 5

Description: This movie demonstrates the active particles can be used to locally increasing the passive particles concentration, thus leading to crystal nucleation. $\phi_a < 0.5\%$ and $\phi_p \approx 35\%$. The movie is accelerated 4X.
